# The association between different impact exercises and osteoporosis: an analysis of data from the Taiwan biobank

**DOI:** 10.1186/s12889-024-19403-y

**Published:** 2024-07-15

**Authors:** Min-Chen Wu, Oswald Ndi Nfor, Chien-Chang Ho, Wen-Yu Lu, Yung-Po Liaw

**Affiliations:** 1 Office of Physical Education, Chung Yuan Christian University, Taoyuan City, 320314 Taiwan; 2 Department of Public Health, Institute of Public Health, Chung Shan Medical University, Taichung City, 40201 Taiwan; 3 Department of Medical Imaging, Chung Shan Medical University Hospital, Taichung City, 40201 Taiwan; 4 Department of Physical Education, Fu Jen Catholic University, New Taipei City, 242062 Taiwan

## Abstract

Osteoporosis is a prevalent condition marked by reduced bone density and an elevated risk of fractures, especially among postmenopausal women. Exercise plays a crucial role in preventing and managing osteoporosis, with weight-bearing and impact exercises being particularly effective in enhancing bone density and mitigating disease risk. This study investigated the relationship between various types of impact exercises and osteoporosis using data from the Taiwan Biobank (TWB). The study sample comprised 5,123 individuals without osteoporosis and 1,770 individuals with the condition. Student’s t-test and logistic regression analyses were utilized to assess the associations between exercise types and osteoporosis risk. Results indicated that high-impact exercise significantly reduced the likelihood of developing osteoporosis compared to no exercise (odds ratio; OR = 0.573, 95% CI: 0.406–0.810, *P* = 0.002). Conversely, low-impact exercises did not show a significant overall association with osteoporosis (OR = 1.160, 95% CI: 0.932–1.445, *P* = 0.184). Stratified analysis by sex revealed that high-impact exercise was protective against osteoporosis in men (OR = 0.391, 95% CI: 0.202–0.755, *P* = 0.005), but not significantly so in women (OR = 0.671, 95% CI: 0.438–1.027, *P* = 0.066). These findings suggest that high-impact exercise is associated with a reduced risk of osteoporosis, particularly among Taiwanese men aged 30 to 70.

## Introduction

Osteoporosis, a systemic skeletal disorder characterized by a decrease in bone mass and deterioration of bone tissue, has become a significant public health concern due to its high prevalence, particularly among the aging population [1]. This condition leads to an increased risk of fractures, which can severely impact the quality of life and impose a substantial economic burden on healthcare systems [2]. To address this issue, it is crucial to identify effective preventive and therapeutic strategies that can help maintain and improve bone health.

Exercise is widely recognized as an essential component of a healthy lifestyle, and its role in promoting bone health has been the subject of numerous studies [3]. However, the specific association between different types of exercise and osteoporosis remains unclear. Low-impact exercises, such as walking, swimming, and yoga, are often recommended for individuals at risk of osteoporosis or those already diagnosed with this condition [4]. These exercises are characterized by gentle and fluid movements that exert minimal stress on the joints and bones, reducing the risk of injury. In contrast, high-impact (ground-impact) exercises, involve more vigorous activities such as running, jumping, and weightlifting, which generate greater forces on the bones and may potentially enhance bone density [5].

The existing literature presents controversial results, with some studies suggesting that high-impact exercises are more effective in preventing and treating osteoporosis [5], while others argue that low-impact exercises can provide similar benefits with a lower risk of injury [4]. These discrepancies may be attributed to differences in study populations, methodologies, and definitions of low- and high-impact exercises. further research is necessary to gain a better understanding of the association between exercise type and osteoporosis, particularly in the context of the Taiwanese population.

The Taiwan Biobank offers a unique opportunity to clarify these associations by providing a large, diverse, and well-characterized sample of Taiwanese individuals. Analyzing data from the biobank offers valuable insights into the association between low-impact and high-impact exercises and the prevalence of osteoporosis. This study aimed to investigate the relationship between low-impact and high-impact exercises and the prevalence of osteoporosis using data from the TWB. By exploring the potential benefits and risks of various types of exercise, this research can help create evidence-based guidelines to promote bone health and alleviate the burden of osteoporosis in Taiwan and beyond.

## Materials and methods

### Study population

This study analyzed data from the TWB, collected from 2016 to 2020. The initial sample included 20,391 biobank volunteers aged 30–70 who had dual-energy X-ray absorptiometry (DXA) measurement data. Participants who engaged in less than 90 min of exercise each week (*n* = 423) and women without information on menopausal status (*n* = 6) were excluded, resulting in 19,962 participants. Additionally, individuals with bone mineral density (BMD) T-scores between − 2.5 and − 1.0 were excluded (*n* = 12,060). We also excluded participants with missing values for any variables in the logistic regression analysis (*n* = 1,009). This refinement process resulted in a final sample of 1,770 osteoporosis cases and 5,123 non-osteoporosis controls. Informed consent was obtained from all volunteers participating in the TWB project. The study was approved by the Chung Shan Medical University’s Institutional Review Board (IRB), with reference number CS1-20009.

### Variable definition

The identification of osteoporosis was based on the participant’s BMD measured at the total femoral sites using DXA scans (DiscoveryTM QDRTM Bone Densitometry Systems (HOLOGIC) machine). Participants with T-scores ≤ -2.5 were categorized as having osteoporosis, while T-scores between − 1.0 and − 2.5 indicated osteopenia. Those with a T-score ≥ -1.0 were considered normal. The analysis encompassed several types of exercise categorized as no exercise (defined as being sedentary or not meeting the recommended level of at least 15 min per day or 90 min per week) [6], low impact exercise (defined as activities where at least one foot remains on the ground at all times, such as walking, swimming, cycling, yoga, and pilates), and high impact exercise (such as jogging, running, jumping jacks, box jumps, jump squats, and burpees), where both feet leave the ground simultaneously, resulting in a forceful impact when landing. Covariates such as age, cigarette smoking (regular smoking for over six months), alcohol intake (consuming at least 150 mL of alcohol per week for more than six months), BMI categories (measured as weight in kilograms divided by height in meters squared), vegetarian diet (adhering to a vegetarian diet for over six months), coffee intake (minimum of three times per week), and weekly exercise duration (average duration per week), were incorporated into the regression model. These features have been previously evaluated for their association with osteoporosis risk [7–9].

### Statistical analysis

Data analysis was performed using SAS software (version 9.4; SAS Institute, Cary, NC, USA). Student’s t-test was employed to examine differences between continuous variables. The Chi-square test assessed differences between categorical variables. Categorical variables were reported as numbers and percentages, while continuous variables were presented as means ± standard errors (SEs). Logistic regression was utilized to investigate the association between exercise type and osteoporosis. The adjusted variables included age, cigarette smoking, sex, alcohol intake, BMI categories, vegetarian diet, coffee intake, mode of living, and weekly exercise duration. The statistical significance threshold was established at *p* < 0.05.

## Results

The study population consisted of 5,123 individuals without osteoporosis and 1,770 individuals with osteoporosis. Significant differences were observed between these groups across multiple demographic and lifestyle variables (Table 1). Those with osteoporosis were less likely to engage in high-impact exercise, with only 4.86% participating compared to 10.33% of those without osteoporosis. Additionally, osteoporosis was more prevalent among women, comprising 83.67% of the affected group, in contrast to 59.91% in the non-osteoporotic group. The age distribution showed that the prevalence of osteoporosis increased with age, particularly among those aged 61 years and older, who represented 61.95% of the osteoporotic population.

**Table 1:** Demographic characteristics of the study population, stratified according to the presence or absence of osteoporosis

Variables	No osteoporosis	Osteoporosis	*P*-value
	(*N* = 5123)	(*N* = 1770)	
Exercise type			< 0.001
No exercise	3087 (60.26)	804 (45.42)	
Low-impact exercise	1507 (29.42)	880 (49.72)	
High-impact exercise	529 (10.33)	86 (4.86)	
Sex, n (%)			< 0.001
Women	3069 (59.91)	1481 (83.67)	
Men	2054 (40.09)	289 (16.33)	
Age, years, n (%)			< 0.001
30–40	982 (19.17)	52 (2.94)	
41–50	1687 (32.93)	92 (5.20)	
51–60	1445 (28.21)	533 (30.11)	
≥ 61	1009 (19.70)	1093 (61.95)	
Cigarette smoking, n (%)			< 0.001
No	3992 (77.92)	1609 (90.90)	
Yes	1131 (22.08)	161 (9.10)	
Alcohol intake, n (%)			< 0.001
No	4450 (86.86)	1675 (94.63)	
Yes	673 (13.14)	95 (5.37)	
BMI categories, n (%)			< 0.001
Underweight (BMI < 18.5 kg/m^2^)	45 (0.88)	165 (9.32)	
Normal weight (18.5 ≤ BMI < 24 kg/m^2^)	1643 (32.07)	1199 (67.74)	
Overweight (24 ≤ BMI < 27 kg/m^2^)	1676 (32.72)	302 (17.06)	
Obese (BMI ≥ 27 kg/m^2^)	1759 (34.34)	104 (5.88)	
Vegetarian diet, n (%)			< 0.001
No	4749 (92.70)	1542 (87.12)	
Yes	374 (7.30)	228 (12.88)	
Coffee intake, n (%)			< 0.001
No	2663 (51.98)	1137 (64.24)	
Yes	2460 (48.02)	633 (35.76)	
Weekly exercise duration (mean ± SE)	103.70 ± 2.40	136.00 ± 4.00	< 0.001
Mode of living			0.789
Country-side dwelling (ref)	50 (0.98)	16 (0.90)	
Urban dwelling	5073 (99.02)	1754 (25.68)	
Menopausal status			< 0.001
No	1862 (60.67)	95 (6.41)	
Yes	1207 (39.33)	1386 (93.59)	

n, sample size; SE, standard error; %, percent; BMI, body mass index; kg, kilogram; m^2^, meter squared

Lifestyle factors also showed notable differences. A smaller percentage of individuals with osteoporosis smoked cigarettes (9.10% vs. 22.08% in the non-osteoporotic group), and fewer consumed alcohol (5.37% vs. 13.14%). In terms of body mass index (BMI), those with osteoporosis were more likely to be underweight and less likely to be obese. Specifically, 9.32% of the osteoporotic group were underweight compared to just 0.88% of those without osteoporosis, while only 5.88% of those with osteoporosis were obese, compared to 34.34% without osteoporosis. Dietary habits revealed that a higher percentage of individuals with osteoporosis followed a vegetarian diet (12.88% vs. 7.30%), and a larger proportion did not consume coffee (64.24% vs. 51.98%).

The Student’s t-test analysis indicated significant differences in ground impact exercise across different BMD categories (Table 2). Compared to those with normal BMD, a higher prevalence of low-impact exercise (49.72%) was observed among the osteoporotic group. High-impact exercise was less common among those with osteoporosis compared to those with normal BMD.

**Table 2:** Results of Student’s t-test analysis, examining the relationship between ground impact exercise and osteoporosis status, with participants categorized into normal bone mineral density, osteopenia, and osteoporosis groups

Variables	Normal BMD	Osteopenia	Osteoporosis	*P*-value
	(*N* = 5123)	(*N* = 12,060)	(*N* = 1770)	
Exercise type				< 0.001
No exercise	3087 (60.26)	6511 (53.99)	804 (45.42)	
Low-impact exercise	1507 (29.42)	4715 (39.10)	880 (49.72)	
High-impact exercise	529 (10.33)	834 (6.92)	86 (4.86)	

Abbreviation: BMD = bone mineral density

Logistic regression analysis further highlighted significant associations between various study variables and osteoporosis (Table 3). High-impact exercise was associated with a lower risk of osteoporosis, with an OR of 0.573 (95% CI: 0.406–0.810, *P* = 0.002), indicating a protective effect. Men had a lower risk of osteoporosis than women (OR = 0.301, 95% CI: 0.245–0.370, *P* < 0.001). The risk of osteoporosis increased substantially with age, particularly for individuals aged 61 and older (OR = 41.657, 95% CI: 29.492–58.840, *P* < 0.001). Higher BMI categories were protective against osteoporosis, with obesity showing the strongest protective effect (OR = 0.010, 95% CI: 0.006–0.015, *P* < 0.001). A vegetarian diet was associated with a higher risk of osteoporosis (OR = 1.718, 95% CI: 1.348–2.191, *P* < 0.001), while coffee intake was linked to a reduced risk (OR = 0.735, 95% CI: 0.635–0.851, *P* < 0.001). A significant interaction was also observed between impact exercise and sex (*P*-value < 0.001).

**Table 3:** The association between osteoporosis and the study variables, with a particular emphasis on the relationship with ground impact exercise

Variables	OR	95% CI	*P*-value
Exercise type			
No exercise (ref)	-	-	-
Low-impact exercise	1.160	0.932–1.445	0.184
High-impact exercise	0.573	0.406–0.810	0.002
P for trend	*P*-value = 0.0331		
Sex			
Women (ref)	-	-	-
Men	0.301	0.245–0.370	< 0.001
Age			
30–40 (ref)	-	-	-
41–50	1.154	0.790–1.685	0.458
51–60	10.026	7.162–14.036	< 0.001
≥ 61	41.657	29.492–58.840	< 0.001
Cigarette smoking			
No (ref)	-	-	-
Yes	1.032	0.798–1.335	0.809
Alcohol intake			
No (ref)	-	-	-
Yes	0.709	0.530–0.950	0.021
BMI categories			
Underweight (ref)	-	-	-
Normal weight	0.134	0.087–0.205	< 0.001
Overweight	0.029	0.019–0.045	< 0.001
Obese	0.010	0.006–0.015	< 0.001
Vegetarian diet			
No (ref)	-	-	-
Yes	1.718	1.348–2.191	< 0.001
Coffee intake			
No (ref)	-	-	-
Yes	0.735	0.635–0.851	< 0.001
Weekly exercise duration	0.999	0.998-1.000	0.001
Mode of living			
Country-side dwelling (ref)	-	-	-
Urban dwelling	0.705	0.327–1.518	0.3714
Interaction of ground impact and sex	*P*-value < 0.0001		

BMI, body mass index; kg, kilogram; m^2^, meter squared

When stratified by sex, the analysis revealed that high-impact exercise was protective against osteoporosis in men (OR = 0.391, 95% CI: 0.202–0.755, *P* = 0.005) but not significantly so in women (OR = 0.671, 95% CI: 0.438–1.027, *P* = 0.066) (Table 4). The risk of osteoporosis increased with age in both sexes, but the magnitude was higher in women aged 61 years and older (OR = 57.177, 95% CI: 38.633–84.623, *P* < 0.001) compared to men (OR = 14.512, 95% CI: 6.949–30.306, *P* < 0.001). Cigarette smoking and alcohol intake were not significantly associated with osteoporosis in either sex.

**Table 4:** The analysis of the association between ground impact exercise and osteoporosis, stratified by the sex of the participants

Variables	Women			Men		
	OR	95% CI	*P*-value	OR	95% CI	*P*-value
Exercise type						
No exercise (ref)	-	-	-	-	-	-
Low-impact exercise	1.120	0.863–1.454	0.393	1.180	0.746–1.867	0.479
High-impact exercise	0.671	0.438–1.027	0.066	0.391	0.202–0.755	0.005
P for trend	*P* = 0.292			*P* = 0.011		
Age						
30–40 (ref)	-	-	-	-	-	-
41–50	0.837	0.541–1.295	0.424	2.986	1.359–6.561	0.007
51–60	10.918	7.514–15.864	< 0.001	5.671	2.662–12.079	< 0.001
≥ 61	57.177	38.633–84.623	< 0.001	14.512	6.949–30.306	< 0.001
Cigarette smoking						
No (ref)	-	-	-	-	-	-
Yes	1.336	0.838–2.129	0.224	1.042	0.764–1.420	0.797
Alcohol intake						
No (ref)	-	-	-	-	-	-
Yes	0.750	0.462–1.219	0.246	0.694	0.481–1.003	0.052
BMI categories						
Underweight (ref)	-	-	-	-	-	-
Normal weight	0.151	0.094–0.244	< 0.001	0.069	0.023–0.204	< 0.001
Overweight	0.032	0.019–0.052	< 0.001	0.014	0.005–0.041	< 0.001
Obese	0.010	0.006–0.017	< 0.001	0.005	0.001–0.015	< 0.001
Vegetarian diet						
No (ref)	-	-	-	-	-	-
Yes	1.589	1.198–2.107	0.001	2.339	1.418–3.859	0.001
Coffee intake						
No (ref)	-	-	-	-	-	-
Yes	0.787	0.662–0.935	0.007	0.611	0.451–0.827	0.001
Weekly exercise duration	0.999	0.998-1.000	0.048	0.998	0.997–0.999	0.005
Mode of living						
Country-side dwelling (ref)	-	-	-	-	-	-
Urban dwelling	0.936	0.351–2.499	0.896	0.470	0.148–1.497	0.202

BMI, body mass index; kg, kilogram; m^2^, meter squared

Among women, the relationship between ground-impact exercise and osteoporosis varied by menopausal status (Table 5). High-impact exercise showed a trend towards a protective effect in non-menopausal women (OR = 0.468, 95% CI: 0.136–1.614, *P* = 0.230), although this was not statistically significant. No significant association was observed for high-impact exercise among menopausal women (OR = 0.701, 95% CI: 0.440–1.118, *P* = 0.136).

## Discussion

The current study investigated the relationship between various types of impact exercises and osteoporosis using data from the Taiwan Biobank. One of the most notable findings is the protective effect of high-impact exercises against osteoporosis. Specifically, individuals who engaged in high-impact exercises were significantly less likely to have osteoporosis compared to those who did not exercise, which is consistent with previous studies [10–12]. In contrast, low-impact exercises did not show a significant overall association with osteoporosis. High-impact exercise generates mechanical loading that stimulates bone remodeling and can lead to increased bone density, [13] thereby helping to prevent osteoporosis.

Sex differences were also prominent, with men showing a significantly lower risk of osteoporosis compared to women. This finding aligns with existing literature [14] indicating that men generally have a higher BMD than women, likely due to hormonal differences and body composition factors. The current study’s conclusion is consistent with previous research [11], suggesting that high-impact exercise may offer more significant protective effects against osteoporosis and bone loss in men compared to women. Despite this, previous studies have reported that high-impact exercise significantly improved bone mineral density in women, particularly postmenopausal women with osteopenia [15] or osteoporosis [16–18]. In the current study, high-impact exercise showed a trend toward a protective effect in both menopausal and non-menopausal women, although this was not statistically significant.

Our study also highlights the substantial influence of age on osteoporosis risk. Age stratification plays a significant role in understanding and managing osteoporosis. By recognizing how bone density changes across different age groups, healthcare providers can better identify at-risk individuals and implement effective prevention and treatment strategies. We found in the current study that the risk of osteoporosis increased dramatically with age, particularly for individuals aged 61 and older. This aligns with the well-established understanding that bone mineral density decreases with age, making older adults more susceptible to osteoporosis [19]. Despite the age group, taking proactive steps to maintain bone health is essential for reducing the risk of osteoporosis and ensuring a better quality of life.

It is well-known that certain ethnicities and genetic backgrounds also influence osteoporosis risk. Based on past literature [20–22], osteoporosis is more prevalent among Caucasian populations, particularly in postmenopausal women. Factors contributing to this include lower peak bone mass, hormonal changes, and lifestyle factors such as diet and physical activity levels.

Physical exercise plays a critical role in maintaining and improving BMD across all stages—from normal BMD to osteopenia and osteoporosis. To gain more insights into the role played by exercise, we did group analysis and found that compared to those with normal BMD, a higher prevalence of low-impact exercise was observed among the osteoporotic group whereas high-impact exercise was less common.

Regarding body size composition, we found that a higher BMI was protective against osteoporosis in both men and women. This is potentially due to the mechanical load on bones and higher estrogen levels from adipose tissue. Asians typically have lower BMI, a risk factor for lower BMD. However, this did not increase osteoporosis risk in our study. In addition, a vegetarian diet remained a risk factor for osteoporosis in both men and women.

The environment in which individuals live—urban or rural—can have a significant impact on their risk of developing osteoporosis. Various factors associated with urban and countryside living, such as lifestyle, physical activity levels, diet, and access to healthcare, play important roles in bone health. In the current study, we found that residing in an urban environment was associated with a lower but non-significant risk of osteoporosis compared to living in the countryside. However, the sample size of the countryside dwellers was relatively small, necessitating further investigations with larger samples. Urban dwellers may face challenges related to lower physical activity, limited sunlight exposure, and dietary habits, while rural residents might encounter issues with healthcare access and nutritional variety. Both environments have unique sets of advantages and disadvantages, and understanding these can help tailor osteoporosis prevention and treatment strategies to the specific needs of individuals in different settings.

These findings have several practical implications. Encouraging participation in high-impact exercises could be a crucial strategy in osteoporosis prevention programs, especially for older adults and postmenopausal women who are at higher risk. Tailored exercise programs that consider individual risk factors can optimize bone health outcomes. Additionally, lifestyle modifications should be integral components of osteoporosis prevention and management strategies. Nutritional guidance should also be provided to individuals at risk of osteoporosis.

This study has several limitations that should be considered. First, the TWB questionnaires do not capture data on exercise intensity or specific types of ground-impact exercises, limiting our ability to recommend an optimal exercise program for osteoporosis prevention. Second, the cross-sectional nature of the study prevents establishing a causal relationship between ground-impact exercise and osteoporosis risk. Additionally, the self-reported nature of the exercise data may lead to recall bias or misclassification, though this misclassification is considered non-differential, tending to bias the results toward the null hypothesis. Future longitudinal studies or randomized controlled trials would be essential to confirm our study findings. Furthermore, environmental and industrial substances such as lead, cadmium, phthalates, and per- and poly-fluoroalkyl substances (PFASs) have been linked to osteoporosis [23], but this information was not included in the biobank. Next, the sample size for countryside dwellers was relatively small, necessitating further investigations with larger samples. Finally, we utilized femoral neck T-scores to define osteoporosis, as this was the primary measurement available in the Taiwan Biobank dataset. Vertebral BMD is also an important indicator of osteoporosis but was not assessed in this analysis. Future studies should aim to incorporate both femoral neck and vertebral BMD assessments to provide a more comprehensive evaluation of osteoporosis status in this population.

In conclusion, high-impact exercise was associated with a lower risk of osteoporosis, particularly among men. Combining high-impact exercises with proper nutritional support can maximize the benefits for bone health. Encouraging these exercise regimens in younger women and men can help build stronger bones, reducing the risk of osteoporosis later in life.

**Table 5:** The relationship between ground impact exercise and osteoporosis, stratified according to the menopausal status of the female population

Variables	Non menopausal (*n* = 1957)			Menopausal (*n* = 2593)		
	OR	95% CI	*P*-value	OR	95% CI	*P*-value
Exercise type						
No exercise (ref)	-	-	-	-	-	-
Low-impact exercise	0.628	0.259–1.521	0.303	1.188	0.896–1.576	0.232
High-impact exercise	0.468	0.136–1.614	0.230	0.701	0.440–1.118	0.136
Age						
30–40 (ref)	-	-	-	-	-	-
41–50	0.715	0.438–1.168	0.181	0.117	0.013–1.089	0.060
51–60	1.494	0.758–2.943	0.246	0.590	0.067–5.179	0.634
≥ 61	-	-	-	2.237	0.255–19.628	0.467

BMI, body mass index; kg, kilogram; m^2^, meter squared. Adjusted for smoking, drinking, BMI, diet, coffee intake, weekly exercise duration, and mode of living.

## Data Availability

The data and software underlying the findings of this study are available from the corresponding author upon reasonable request.
